# Design and Development of Antibacterial/Anti-inflammatory Dual Drug-Loaded Nanofibrous Inserts for Ophthalmic Sustained Delivery of Gentamicin and Methylprednisolone: *In Vitro* Bioassay, Solvent, and Method Effects’ Evaluation

**DOI:** 10.34172/apb.2022.056

**Published:** 2021-07-04

**Authors:** Shahla Mirzaeei, Donya Barfar

**Affiliations:** ^1^Pharmaceutical Sciences Research Center, Health Institute, Kermanshah University of Medical Sciences, Kermanshah, Iran.; ^2^Nano Drug Delivery Research Center, Health Technology Institute, Kermanshah University of Medical Sciences, Kermanshah, Iran.; ^3^Student Research Committee, Kermanshah University of Medical Sciences, Kermanshah, Iran.; ^4^Research and Development Department, Rahesh Daru Novin Inc., Kermanshah University of Medical Sciences, Kermanshah, Iran.

**Keywords:** Gentamicin, Nanofibers, Ophthalmic drug delivery, Methylprednisolone, Sustained-release

## Abstract

***Purpose:*** To overcome the challenges caused by the use of conventional ophthalmic dosage forms such as the fast elimination of the drug from the surface of the eye, in this study, dual drug-loaded nanofibers were developed for sustained ophthalmic delivery of gentamicin (GNT) and methylprednisolone (MP). Moreover, the solvent effects, polymer mixtures, and method of preparation on the release profile of the prepared nanofibers, were evaluated.

***Methods:*** The nanofibers were prepared using polycaprolactone (PCL), poly (lactic-co-glycolic acid) (PLGA), and polyvinyl alcohol (PVA) using electrospinning technique. Thereafter, seven optimized formulations were developed with different solvent mixtures and polymer concentrations using various electrospinning methods. The physicochemical and mechanical properties of nanofibers were also evaluated, and the morphology of formulations was observed. The antibacterial efficacy was investigated and the *in vitro* release amounts of GNT and MP from nanofibers were estimated using the bioassay and ultraviolet-visible (UV-Vis) spectroscopy.

***Results:*** The developed G1, G4, G5, G6, and G7 had suitable mechanical properties and morphologies with diameter ranging between 70-350 nm. The 1:1 v/v ratio of DMF/DCM in the solvent mixture and using core-shell technique for the preparation, formed nanofibers with more favorable release profiles. The optimized formulations indicated sustained-release manner for both drugs during 3-9 days and the antibacterial efficacy against *Staphylococcus aureus*.

***Conclusion:*** Among all the prepared formulations, the nanofiber with core-shell structure possessed the best sustained-release profiles of GNT and MP. The obtained results suggest that these nanofibers have a potential to be used as an insert in the eye for long-term release of the drug.

## Introduction

The main challenge in the ophthalmic administration of drugs is the maintenance of a therapeutic concentration at the site of action for longer period. Due to the barrier structure of the eye, which makes it impenetrable, most of foreign substances like drugs cannot enter the different layers of the eye.^1,2^ Rapid elimination of drugs from the surface of the cornea as a result of blinking and fast tear film turnover, would consequently limit the bioavailability and efficacy of topical ophthalmic formulations such as eye drops and ointments.^3^ Despite all these challenges, topical ophthalmic forms have the advantages of reducing the side effects compared to systemic formulations, because of their more targeted delivery of drugs besides being non-invasive and patient’s compliant.^4,5^ Hence, novel ophthalmic delivery systems with sustained release profile and enhanced penetration through the cornea, were designed and introduced in recent years.^3^ The viscosity-enhanced eye drops and water-insoluble ointments are the examples of the modified conventional formulations, which can almost increase the contact time of drugs with the eye. However, they still require being frequently administered during the day.^6^

Polymer-based novel delivery systems are popular dosage forms with the aim of achieving a sustained-release of drugs at the site of action, which can be used in drug delivery, tissue engineering, etc.^7-9^ Mucoadhesive nanoparticles, nanofibers, and polymeric inserts are known as novel drug delivery systems designed and evaluated previously for the delivery of ophthalmic agents.^10,11^ Nanofibers are polymeric drug carriers usually prepared by biodegradable polymers using the electrospinning method.^12^ During the electrospinning process, a high voltage is applied to an injecting polymer solution, which consequently causes elongation of the droplets that can form the nanofibers after the solvent’s evaporation.^13^ Nanofibers have the advantage of high surface to volume ratio, which can eventually enhance the drug bioavailability. Moreover, these systems can increase drug residence time and provide a sustained-release formulation.^14-16^

Ophthalmic superficial conditions, including bacterial infections, blepharitis, conjunctivitis, and corneal ulcers, are commonly managed by the administration of topical dosage forms. Gentamicin (GNT), as an anti-infective agent belongs to the aminoglycoside class, is reported to be effective on various microorganisms such as *Escherichia coli*, *Staphylococcus aureus*, and *Pseudomonas aeruginosa.*^17-19^ *Pseudomonas aeruginosa* is known as a common cause of ophthalmic infections, which can be treated by typical GNT administration.^20^ One of the advantages of GNT is that there is a lower chance of developing drug resistance to it compared to other aminoglycosides.^21^ Methylprednisolone (MP) is a corticosteroid with anti-inflammatory effects, which has a four-time greater potency compared to hydrocortisone. Accordingly, the MP eye drop is administrated for dry eye syndrome.^22,23^ Many commercial products contain either GNT or a corticosteroid, both of which are usually required to be administrated concurrently under some ophthalmic conditions. Designing a multiple-drug delivery system has the advantage of the enhanced patient compliance, due to requiring the administration of only one product, instead of a number of preparations. There is a commercially manufactured product entitled PRED-G®, which contains Prednisolone and GNT in the ointment and suspension forms that can specifically be used in the treatment of superficial bacterial ophthalmic infections and the corresponding inflammation, respectively. Preparing a similar prolonged-release formulation can overcome the other challenges related to the administration of conventional dosage forms, which require frequent administrations of the drug.^24^ Besides, despite the ophthalmic ointments, inserts do not consequently cause blurry vision.^25^

Dextenza® is an insert releasing dexamethasone by passing up to 30 days after being placed in the eye. Mydriasert® and Lacrisert® are the other examples of topical ocular inserts. These systems have various advantages, including sustained-release, prolonged contact time due to the mucoadhesive nature of polymers, and lower elimination rate in comparison to the solution and suspension drops, indicated in previous studies.^26-28^ Accordingly, in a previous study, polymeric ophthalmic inserts containing azithromycin-loaded Eudragit® L100 nanoparticle with suitable physicochemical properties have shown sustained *in vitro* and *in vivo* drug releases.^29^

Considering the advantages of nanofibers and the broad-spectrum effect of GNT on various ophthalmic pathogens, in this study, polycaprolactone (PCL) and poly (lactic-co-glycolic acid) (PLGA) nanofibers were developed using electrospinning technique, in order to achieve the sustained release ophthalmic inserts. The effects of different electrospinning techniques and solvent mixtures on the morphology and release profile of the nanofibers, were evaluated. In addition, we evaluated the physicochemical and mechanical properties of nanofibers. Moreover, the antibacterial efficacy and *in vitro* release of both GNT and MP from nanofibers were estimated using bioassay and ultraviolet-visible (UV-Vis) spectroscopy.

## Materials and Methods

### Materials

GNT was purchased from Acros Organics (Geel, Belgium). MP and PCL were also obtained from Sigma-Aldrich (Milan, Italy). Polyvinyl alcohol (PVA) (99% hydrolyzed, average Mw = 72,000 Da), acetic acid at 100%, Tryptic Soy Agar (TSA), sodium dihydrogen phosphate dodecahydrate, dichloromethane (DCM), and N, N-dimethylformamide (DMF) were purchased from Merk (Darmstadt, Germany). Additionally, PLGA was obtained from Boehringer Ingelheim (Ingelheim, Germany).

### Methods

#### Preparation of electrospinning solutions

#### Preparation of PCL/GNT electrospinning solutions

The solvent mixture containing DCM and DMF was used to dissolve the drug and polymer. Thereafter, three formulations of G1, G2, and G3 with different DCM: DMF ratios of 1:1, 3:2, and 9:1 were developed. For all the formulations, a specific amount of PCL was dissolved in the solvent mixture, in order to prepare a 10% w/v solution for 1 hour under continuous stirring (1000 rpm) at 25°C. Finally, GNT was added to the solution and then stirred to obtain a homogenous solution containing GNT with 10% w/w of the total polymer used during the preparation of formulation (Table 1).

**Table 1:** The concentration of drugs and polymers, solvent mixture, method of preparation, viscosity and conductivity of electrospinning solutions, used for development of formulations

**Components**	**Formulation**						
	**G1**	**G2**	**G3**	**G4**	**G5**	**G6**	**G7**
PCL (%w/v)	10	10	10	10	10	10	0
PVA (%w/v)	0	0	0	15	15	15	10
PLGA (%w/v)	0	0	0	0	0	0	20
MP/Polymer^a^ (%w/w)	0	0	0	6	6	6	6
GNT/Polymer^b^ (%w/w)	10	10	10	20	20	20	20
DCM: DMF	1:1	3:2	9:1	1:1	1:1	1:1	1:1
Viscosity (cP)^c^	150	160	220	120	120	120	100
Conductivity (µs/m)^c^	0.4	0.3	0.1	0.45	0.45	0.45	0.38
Method of preparation	Single-layer	Single-layer	Single-layer	Mixed	Sandwich	Core-shell	Mixed

^a^ The polymer is PCL in case of G4-5 and PLGA in case of G7. ^b^ The polymer is PCL in case of G1-3 and PVA in case of G4-7. ^c^ Measured for organic electrospinning solutions (PCL and PLGA solutions).

#### Preparation of PCL/MP-PVA/GNT electrospinning solution

In order to prepare G4, G5, and G6 formulations, two separate solutions were prepared. PCL was dissolved in DCM: DMF (1:1) to prepare a 10% w/v solution. Thereafter, MP was added (6% w/w of PCL) to the solution for 1 h under magnetic stirring (1000 rpm) at room temperature, in order to obtain a clear PCL/MP solution. Afterward, to prepare the PVA/GNT solution, 15% w/v of PVA was dissolved in distilled water, GNT at a concentration of 20% w/w of the polymer (PVA) was then added to the solution, and continuously stirred (1000 rpm) for 1 hour at 25 °C until obtaining a transparent solution (Table 1).

#### Preparation of PLGA/MP-PVA/GNT electrospinning solutions

At this stage, the G7 formulation was developed using two different solutions. PLGA polymer was dissolved in DCM: DMF (1:1) to obtain a 20% w/v solution, and MP (6% w/w of the PLGA) was then added to this mixture to obtain the PLGA/MP electrospinning solution. To prepare the PVA/GNT solution, 10% w/v of PVA was dissolved in distilled water, and thereafter, GNT with a concentration of 20% w/v of the polymer (PVA) was added to the solution until obtaining a clear solution after 1 h of magnetically stirring (100 rpm) at room temperature (Table 1).

#### Electrospinning procedure

#### Electrospinning procedure of PCL/GNT nanofiber

To prepare the G1, G2, and G3 nanofibers, the single-jet electrospinning process was used. For each formulation, the electrospinning solution was put into a syringe with a 0.1 mm needle, which was then injected toward a rotary collector covered by aluminum foil. A high-voltage supplier was used with an 18 kV voltage to the injected solution. The distance between the injector and collector was adjusted as 10 cm and the whole procedure took place at room temperature. The nanofibers were collected after the evaporation of the solvents for an overnight.

#### Electrospinning procedure of PCL/MP-PVA/GNT nanofiber

The G4 nanofiber was developed with a sandwich structure using the single-jet electrospinning process under the electrospinning conditions similar to those of the G1-3 formulations. A cycle of consecutive initial PVA/GNT layer electrospinning was performed followed by PCL/MP layer as well as another PVA/GNT layer electrospinning. Subsequently, the double-jet electrospinning was established for the preparation of the G5 formulation with a mixed structure. Each one of the prepared solutions of PCL/MP and PVA/GNT was separately filled in a nozzle and then electrospun concurrently by a rotary collector. Correspondingly, the electrospinning condition was similar to that of the G1-3 formulation. The nanofiber was also collected after the complete evaporation of the solvents. The G6, as core-shell nanofiber, was developed by a single-jet electrospinning under electrospinning conditions similar to those of the G1 formulation. As well a core-shell needle was utilized and the PCL/MP solution was put into the core reservoir, while the PVA/GNT solution was put into and injected by the shell injector. The nanofiber was collected after the evaporation of the solvents.

#### Electrospinning procedure of PLGA/MP-PVA/GNT nanofiber

The G7 formulation was prepared using a mixed structure by the double-jet electrospinning. Thereafter, each one of the PLGA/MP and PVA/GNT was filled in an injector and then electrospun concurrently toward the collector under the above-mentioned electrospinning condition. The nanofiber was collected after evaporating the solvents.

#### Morphology characterization

Scanning electron microscopy (SEM) is a typical method used for the characterization of surface morphology, especially in polymeric structures. In this study, the surface properties of the developed nanofibers after became gold-coated were observed by a scanning electron microscope (KYKY, EM-6200, China). The diameter of these nanofibers was calculated for 50 individual strands using the ImageJ software (ImageJ, National Institutes of Health, USA). Of note, the mean ± SD was reported as the average diameter.

#### Fourier-transform infrared spectroscopy (FTIR)

To evaluate the possible interactions occurring between the drug and polymers during the preparation process, FTIR was utilized. In this method, the structure of a sample is analyzed using the specific peaks appearing in the FTIR spectrum, which act like fingerprints in predicting the structure of the compound. The FTIR spectra of GNT, MP, PVA, PCL, and PLGA were also obtained using the KBr pellet method. The FTIR spectrophotometer (Prestige-21, Shimadzu, Nakagyo-ku, Japan) recorded the spectra within the range of 4000 to 400 cm^-1^.

#### Physicochemical characterization

#### Physicochemical properties of electrospinning solutions

Viscosity and conductivity of the electrospinning solutions were measured three times using both rheometer (Brookfield DV-III Ultra programmable, Brookfield Engineering Laboratories, MA, USA) and inoLab® Cond 7110 conductivity meter (WTW GmbH, Weilheim, Germany), respectively. Notably, the mean was reported.

#### Mechanical properties of nanofibers

The folding endurance and thickness of electrospun mats were examined. The folding endurance, which defines the flexibility of nanofibers, can be described as the number of times that a nanofiber is folded at the same point with no tearing and breaking. At this stage, 3×3 patches were repeatedly cut and folded at a specific point until tearing. Thereafter, the average was reported as folding endurance.

Three different points of the nanofibers were examined for the thickness using a digital micrometer (Syntek, Zhejiang, China) with an accuracy of 0.001 mm. In this regard, the mean ± SD was also reported.

#### Physicochemical properties of nanofibers

Drug content uniformity, swelling percentage, moisture loss and uptake, and stability were evaluated for all the prepared nanofibers. Three different samples of each formulation were completely dissolved in their solvents and continuously stirred (1000 rpm) for 1 hour. subsequently, the contents of both GNT and MP were measured using bioassay and UV spectroscopy, respectively.

The swelling percentage was calculated using the standard formula (equation 1). Initially, the nanofibers were weighed, immersed in phosphate buffer solution (PBS) (pH = 7.4), and then re-weighed at intervals of 30, 60, and 120 min.


(Equation 1) $$\begin{array}{l} Swelling\;percentage=\frac{W_{t}-W_{0}}{W_{0}}\times 100\\ W_{t}=Measured\;weight\;at\;time\;t\\ W_{0}=Initial\;weight \end{array}$$

In order to measure the moisture loss and uptake amounts, nanofibers were initially weighed and then placed in desiccators containing anhydrous calcium chloride and aluminum chloride. By passing 72 hours, the samples were re-weighed. The moisture loss and uptake amounts were also measured using the standard formula (equation 2). Three samples were examined for each formulation and an average was then taken.


(Equation 2) Moisture loss and uptake 00=W0−WfW0×100Wf=Final measured weightW0=Initial weight

The long-term stability levels of G1, G2, and G3 nanofibers were evaluated in terms of the International Conference on Harmonization (ICH) guideline. The prepared nanofibers were kept for 12 months at room temperature with 75 ± 5% humidity in aluminum packaging. As well, any change in physicochemical properties was recorded.^30^

#### Antimicrobial efficacy

The McFarland standard suspension of *S. aureus* was prepared and then spread uniformly on TSA. Next, pieces of formulations (1×1 cm^2^) were cut, placed on the plates, and then incubated for 24 hours at 37 °C. Finally, the plates were observed for the inhibition growth zone.

#### Sterility test

To examine the sterility of the formulations, the blank nanofibers were transferred to Thioglycolate broth, Soybean-casein digest broth, and Sabouraud dextrose broth, and then incubated at 37°C. Thereafter, these were observed for any turbidity or growth of anaerobic bacteria, aerobic bacteria, and fungi, after 7, 14, and 28 days, respectively. Positive and negative controls were also prepared for each one of the mediums.

#### Bioassay

Bioassay or microbial assay is a quantification method used for anti-infective agents like GNT. Based on the recently performed studies, this method has the advantage of being more functional for the quantification of antibiotics compared to some common methods such as High-performance liquid chromatography. Additionally, Bioassay has a lower limit of detection (LOD) for the quantification of GNT compared to UV analysis. In the current study, to construct a calibration curve, a McFarland standard bacterial suspension of *S. epidermidis* was prepared and then spread uniformly on TSA plates. Moreover, different standard solutions of GNT (S1 = 500, S2 = 250, …, SS9 = 3.9 µg/mL) were prepared. Subsequently, small wells were punched on the TSA plates, filled with 50 µL of standards, and incubated for 24 hours at 37°C. Accordingly, the whole procedure was repeated three times and an average was also taken. The inhibition growth zones of the wells were measured using Vernier caliper (accuracy = 0.005 mm). As well, the calibration curve was drawn for the mean diameter of inhibition growth zones versus log_10_ of concentrations. Finally, the samples were examined using the same well diffusion method and the regression equation was used to both quantify and estimate the drug’s concentration.

#### UV-Vis spectroscopy

At this stage, a calibration curve for UV-Vis absorption of MP (λ max = 241 nm) against the concentrations, were constructed using the standard solutions of MP (S1 = 100, S2 = 50, …, S10 = 0.195 µg/mL). The obtained regression equation can be used to estimate the drug’s concentration in further studies.

#### In vitro release study

To analyze the *in vitro* release of both GNT and MP from the developed formulations, a device was designed. Each formulation along with 2 mL of PBS (pH = 7.4) were placed in a both-side closed dialysis bag and then immersed in a falcon tube containing 48 mL of PBS as the receptor medium. Correspondingly, the mixture was stirred with 100 rpm at 37°C. The sampling process (1 mL) was performed at different time intervals. To maintain the sink condition, the withdrawn sample at each time was replaced with the same amount of fresh PBS. The amount released GNT at each time of sampling was calculated using the bioassay method and the released MP was also measured using UV-Vis spectroscopy.

## Results and Discussion

### Preparation of nanofibers

Seven formulations were developed in this study. G1, G2, and G3 were prepared using the hydrophobic polymer, PCL dissolved in DCM/DMF as solvent system. It should be noted that although DMF was able to dissolve PCL in a mixture with DCM, it is an excellent polar solvent with high polarity index and miscibility with water which can dissolve GNT. In preparation of the G4, G5, and G6, because of the addition of MP in the organic phase (PCL solution), according to the salting-out, GNT was not allowed to dissolve to the extent same as G1 formulation into the organic phase, therefore, two separate organic and the aqueous phase were prepared. GNT was added to PVA solution in water and MP was added to PCL solution in DCM/DMF. The same process was performed for G7 formulation. The prepared formulations were successfully electrospun and the collected nanofibers were found to have suitable visual and mechanical properties. All these nanofibers were uniform, strong, and flexible, which could be separated from the aluminum foil. Hence, all the formulations were subjected to further studies. The PVA-containing layer were chosen as the outer layer in the formulations because of mucoadhesive nature to enhance the contact time to the ocular tissue.

### Morphology characterization

**Figure 1:**
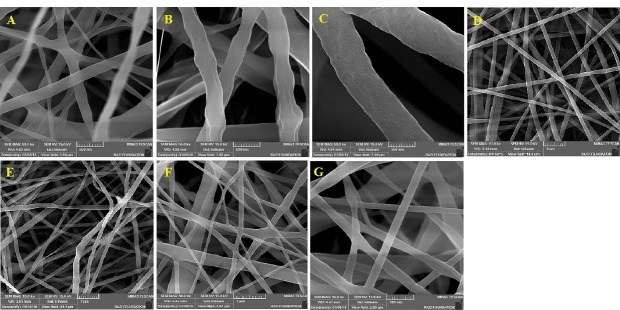
SEM images obtained for different formulations. A: G1, B: G2, C: G3, D: G4, E: G5, F: G6, G: G7.

All the formulations were found to have a uniform structure with no significant beads or defects. The SEM images are shown in Figure 1. The mean diameter of fibers in these different formulations was measured ranged from70 to 650 nm (Table 2). Considering the findings of the previous studies in this regard, the molecular weight, polymer concentration, polymeric solution conductivity, polymeric solution viscosity, and the solvent system can be named as the key parameters affecting the morphology and diameter of nanofibers.^31^ As mentioned earlier, the present study aimed to evaluate the effects of the solvent system, polymeric mixture, and method of preparation on the characteristics of the prepared nanofibers. Among the PCL-GNT formulations (G1, G2, and G3), the least diameter was found to belong to the G1 formulation with a 1:1 ratio of DCM/DMF, while the highest diameter was related to G3. The reason behind this increase in diameter may be the decreased DMF in the solvent system, which led to the decreased conductivity and increased viscosity.^32^ Notably, the increased viscosity consequently led to the increased surface tension of polymeric solution, hence the polymer droplet resisted against being stretched, which caused the formation of fibers along with increasing the diameter. The decreased conductivity resulted in the incapability of the polymeric solution to pass the electric current and stretch the droplets. Therefore, the 1:1 DCM/DMF ratio was chosen as the optimized solvent system for the preparation of other formulations. Despite using different methods for preparation, G4 and G5 were found to have almost the same diameters due to the same conductivity and viscosity of the electrospinning solution. The lower diameter of fibers in the G6 formulation compared to G4 and G5 confirmed the fact that the method of preparation can affect the physicochemical properties of formulations. The fibers with smaller diameters were obtained by core-shell structure. Of note, G7 possessed a mean diameter of less than 150 nm.

**Table 2:** The physicochemical characteristic of different formulations (n = 3, Mean ± SD)

**Parameter**		**Formulation**						
		**G1**	**G2**	**G3**	**G4**	**G5**	**G6**	**G7**
Diameter (nm)		71 ± 29	273 ± 67	620 ± 74	354 ± 63	306 ± 43	158 ± 25	145 ± 82
Folding endurance (times)		256 ± 8	289 ± 4	285 ± 8	416 ± 3	430 ± 5	142 ± 7	306 ± 9
Thickness (mm)		0.076 ± 0.002	0.083 ± 0.002	0.092 ± 0.004	0.099 ± 0.001	0.072 ± 0.002	0.050 ± 0.002	0.045 ± 0.002
Swelling (%)	30 min	18.18 ± 0.05	36.49 ± 0.21	4.31 ± 0.01	8.77 ± 0.02	3.42 ± 0.01	8.74 ± 0.04	7.64 ± 0.05
	60 min	29.54 ± 0.06	49.87 ± 0.11	23.74 ±‌0.10	14.00 ± 0.04	9.58 ± 0.06	26.90 ± 0.06	24.00 ± 0.01
	120 min	50.00 ± 0.12	64.45 ± 0.21	46.47 ± 0.08	96.49 ± 0.25	98.00 ± 0.21	68.00 ± 0.08	29.10 ± 0.09
Moisture loss (%)		2.271 ± 0.005	0.263 ± 0.003	3.333 ± 0.003	1.812 ±0.005	2.914 ±0.008	3.575 ±0.003	2.944 ±0.005
Moisture uptake (%)		3.233 ± 0.008	0.574 ±0.002	0.487 ± 0.007	0.510 ± 0.003	1.498 ± 0.007	2.175 ± 0.005	2.776 ± 0.002

### Fourier-transform infrared spectroscopy

**Figure 2:**
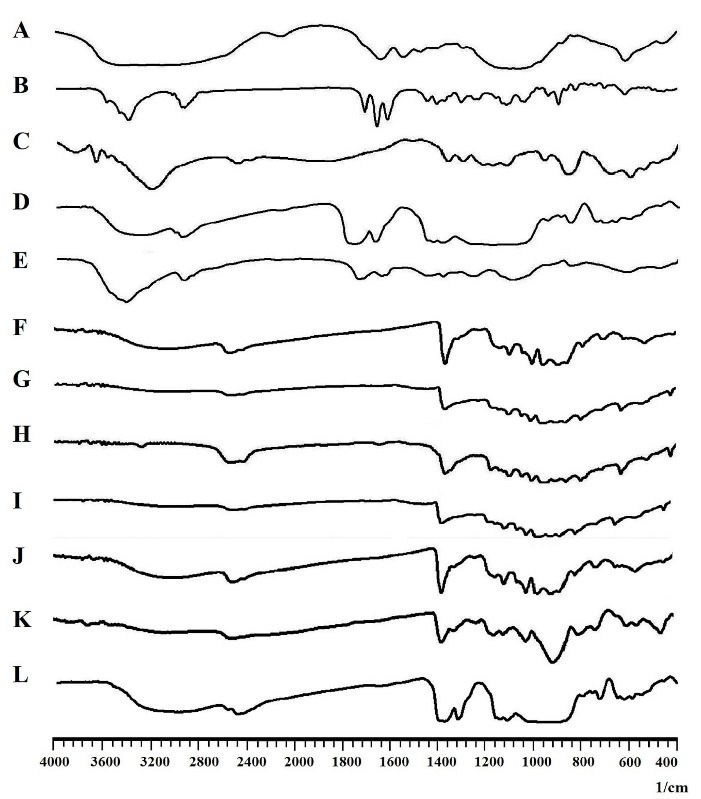
The FTIR spectra of A: Gentamicin, B: Methylprednisolone, C: PCL, D: PLGA, E: PVA, and F: G1, G: G2, H: G3, I: G4, J: G5, K: G6, L: G7 nanofibers.

Figure 2 shows the obtained FTIR spectra of drugs, polymers, and the developed nanofibers.

*G1, G2, and G3:* The characteristic peaks of both PCL and GNT can be observed with a slight shift in the FTIR spectra obtained for G1, G2, and G3. The peak at 3433 cm^-1^ can be attributed to the OH group of PCL and the peaks observed at 2866 and 2935 cm^-1^ can be assigned to the symmetric and asymmetric stretching of C-H bonds in the PCL, respectively. As well, there was a peak at 1724 cm^-1^ related to the conjugated ester groups (C = O) of PCL. The peak at 1169 cm^-1^ was also assigned to stretching of the C-O-C bands. Moreover, the characteristic peaks of GNT can be attributed to the amide bands, bending vibration of S-O, and stretching vibration of S-O, which appeared at 1627, 617, and 1057 cm^-1^, respectively.^33,34^

*G4, G5, and G6:* All the characteristic peaks of PCL and GNT, which appeared at the FTIR spectra of G1, G2, and G3, were also observed with a slight shift for these formulations. The characteristic peaks of MP detected at 1450 and 1369 cm^-1^ can also be related to the CH_3_ bands. In addition, the peaks at 2935, 1440, and 1378 cm^-1^ were attributed to the OH, CH_2_, and CH groups of PVA, respectively.^22,35^

*G7:* Along with all the characteristic peaks of GNT, MP, and PVA appeared in the FTIR spectrum of G7, the peaks at 2993, 2943, and 1099 cm^-1^ were attributed to CH_2_, CH, and C-O groups of PLGA, respectively.^36^

### Physicochemical characterization

#### Physicochemical properties of electrospinning solutions

The measured viscosity and conductivity of polymeric solutions are shown in Table 1. Clearly, the decreased DMF/DCM ratio at the fixed polymer concentration, resulted in the decreased conductivity and increased viscosity, which are not desirable for the electrospinning process.^32^ Because of the higher levels of DMF, G1 had the least viscosity as well as the most conductivity among PCL-GNT formulations. Moreover, because of the same polymer concentration, besides the same solvent system, G4, G5, and G6 were found to have the same levels of conductivity and viscosity for the obtained organic electrospinning solutions (PCL and PLGA solutions). The organic solution in the preparation of G7 formulation showed lower viscosity compared to G4, G5, and G6 formulations and almost the same conductivity to them.

#### Mechanical properties of nanofibers

All the formulations showed uniform thickness across the nanofibrous mats. The mean thickness of the formulations was within the range of 0.043 to 0.099 mm, which is considered as suitable for being applied as ophthalmic inserts. As well, it can be said that the lower thickness of these formulations compared to ophthalmic inserts prepared by the casting method, makes these formulations appropriate for being placed in the conjunctival sac.

The least folding endurance was observed for G6 (142 ± 7) prepared using the core-shell method, while the most folding endurance belonged to G5 (430 ± 5) and G4 (416 ± 3) with the sandwich and mixed structures, respectively. Additionally, G1, G2, and G3 showed the same folding endurance almost around 250, and G7 had folding of 306 ± 9 (Table 2). Owing to the high potential of PCL, PVA, and PLGA to form flexible and strong nanofibers, all the formulations showed a suitable folding endurance for ophthalmic application, which is usually considered to be between 200 and 300.^37^

#### Physicochemical properties of nanofibers

The G1, G4, and G7 formulations with organic solvent ratio of 1:1, showed a uniform drug’s content across all the mats, while the G2 and G3 formulations showed varied contents at different points of mats. Accordingly, this may be due to the lower concentrations of DMF in the preparation of these formulations.

The swelling percentage has a great impact on both the amount and profile of drug release from the nanofibers.^38^ The swelling percentage after 120 minutes is ranged from 29 to 98%. Notably, hydrophilic polymers mostly indicated a higher degree of swelling compared to hydrophobic ones. In this study, the G4, G5, and G6 showed higher levels of swelling because of the presence of the hydrophilic PVA nanofibers in their structure compared to the G1, G2, and G3, which were prepared only by the hydrophobic polymer PCL. The G6 nanofiber prepared using the core-shell method, showed the least swelling among the PCL-MP/PVA-GNT nanofibers. The least swelling percentage was calculated to be belonged to the G7 formulation, which can be due to the higher hydrophobicity of PLGA compared to PCL.^39^

None of the formulations showed more than 4% of moisture loss or uptake amounts after 72 hours. So, based on this finding, it can be concluded that the prepared formulations are capable of remaining as physiochemically stable under dry and humid conditions (Table 2).^40^

The long-term stability studies indicated no significant changes in color, texture, flexibility, and other physicochemical properties of formulations along with almost the same drug content during this period.

### Antimicrobial efficacy

**Figure 3:**
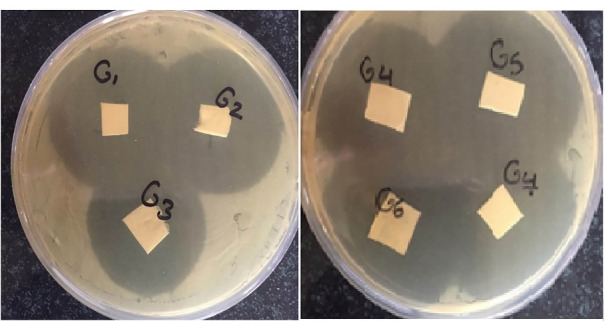
The antimicrobial efficacy of nanofibers (G1, G2, and G3) against *S. aureus.*

As shown in Figure 3, all the prepared nanofibers containing GNT developed inhibition growth zones against gram-positive bacteria (*S. aureus*). *S. aureus* is known as a common cause of ocular infections with an increasing prevalence in recent years.^41^

### Sterility test

None of the formulations showed any turbidity or any sign of microorganism growth in the culture media. Therefore, it can be concluded that the formulations were sterile and the whole preparation procedure took place under the aseptic condition.

### Bioassay

**Figure 4:**
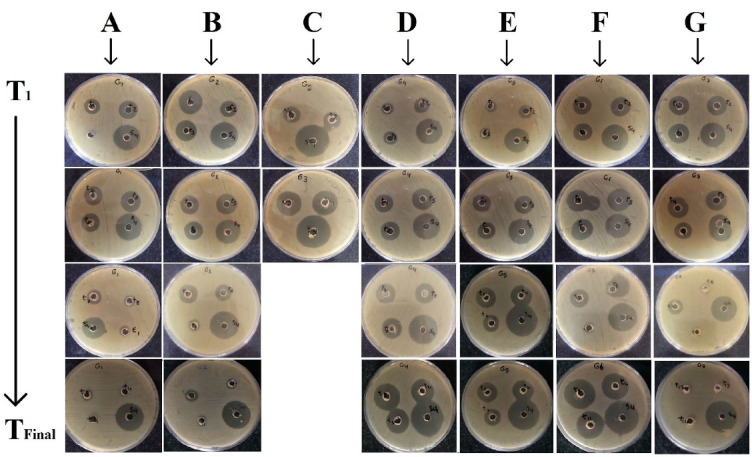
The bioassay incubated plates *in vitro* release samples withdrawn between T_1_ to T_Final_ against *S. epidermidis*, obtained for different formulations. A: G1, B: G2, C: G3, D: G4, E: G5, F: G6, G: G7 nanofibers.

The constructed calibration curve gave the regression equation of y = 1.5675x–0.4268 with an acceptable R^2^ of 0.9856. The amount of GNT should be estimated in further studies, using this equation. No inhibition growth zone was observed for the blank formulations (Figure 4).

### UV-Vis spectroscopy

At this stage, a linear calibration curve was obtained and the regression equation of y = 0.0432x+0.0025 with R^2^ of 0.9984 was calculated. This equation was used to estimate the released MP. It should be noted that all the components of the formulations were examined for having absorption in λ max of MP. As a result, the polymers or GNT had no significant UV absorption at a similar wavelength, which could be due to an error in the assay.

### In vitro release study

**Figure 5:**
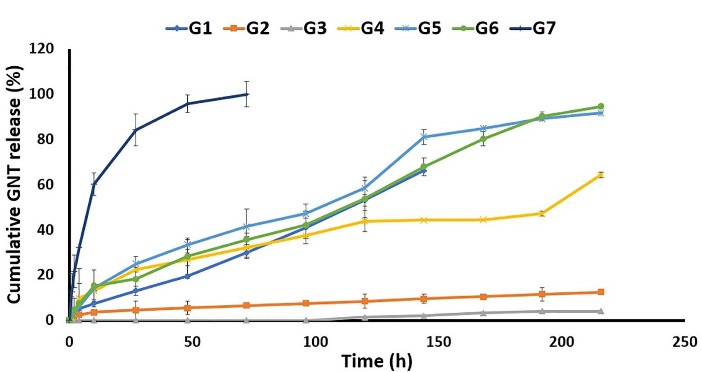
The *in vitro* release profile of Gentamicin from different formulations in PBS (pH = 7.4) at 37 °C.

As shown in Figure 5, G1 released 66.24 ± 0.04% of GNT during 144 h, while G2 and G3 only released 9.55 ± 2.38% and 2.28 ± 0.14% of GNT during 144 h reaching up to almost 12% and 4% release after 216 h, respectively. It should be noted that G3 indicated a delayed release from 120 to 216 hours. Accordingly, the main reason behind the decreased drug release in the G2 and G3 formulations was the lower concentration of DMF in the solvent systems, which led to a lack of uniformity and variable drug content. Apart from the decreased viscosity and enhanced conductivity, which were achieved by the higher amount of DMF in the solvent systems, GNT was found to be more soluble in DMF compared to DCM. As a result, the G1 electrospinning solution, as the most homogenous electrospinning solution, with the optimum DMF: DCM ratio (1:1 v/v) was observed to possess the best electrospinning characteristics. The G1 indicated a suitable release profile by releasing GNT during 144 hours.

**Figure 6:**
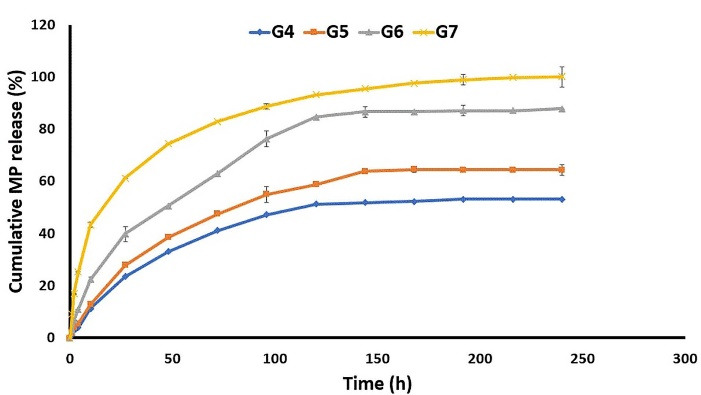
The *in vitro* release profile of Methylprednisolone from different formulations in PBS (pH = 7.4) at 37 °C.

As well, G4 released almost 60% of its GNT content during 216 hours, while releasing about 53% of MP at the same interval. Moreover, G5 released 91.62 ± 0.18 of GNT in 216 h, and 64.40 ± 2.01 of MP in the meanwhile. In addition, the G6 formulation released almost 95% of GNT and 87% GNT in 216 hours (Figure 6). As indicated, the nanofibers prepared by the core-shell structure showed higher levels of the released MP and GNT compared to the mixed and sandwich structures. By comparing the mixed and sandwich structures, it seems that the sandwich structure was better in terms of the released GNT and MP. Finally, it was observed that, among the G4, G5, and G6, the G6 formulation had the best release profile by the prolonged release of both drugs in a 9-day period. The G5 formulation also indicated an almost suitable release profile. So, it could be concluded that the method of preparation is an important factor affecting the release profile of drugs from nanofibers.

The G7 formulation released more than 90% of MP during 216 h, while it released most of its GNT content in 72 h. The release profile of G7 can also be considered as suitable for the short-term treatment of bacterial ophthalmic infections. Similar results were also reported in a previous study, which indicated that polymeric Azithromycin ophthalmic inserts containing Eudragit® L100 nanoparticle could sustain both the *in vitro* and *in vivo* drug release.^29^

## Conclusion

Due to the different challenges of conventional ophthalmic drug delivery, including fast elimination from the surface of the cornea and requiring frequent administrations, novel ophthalmic carriers were recently designed and introduced in this regard. In this essay, dual drug-loaded nanofibers were used for the sustained ophthalmic release of GNT and MP. This study evaluated the effects of the solvent system, polymeric mixture, and method of preparation on the characteristics of the prepared nanofibers. So, seven formulations were prepared using PCL, PVA, and PLGA using electrospinning technique. It was observed that among different solvent systems examined, the 1:1 solvent mixture of DCM and DMF obtained the best nanofibers (G1), because of the enhanced viscosity and conductivity levels along with more electrospinning solution uniformity. Moreover, the G1, G4, G5, G6, and G7 nanofibers showed suitable mechanical and physicochemical properties along with antimicrobial efficacy against *S. aureus*. The G6 formulation with the core-shell structure showed the best release profile by the prolonged release of both drugs in a 9-day period. The G5 and G7 formulations also indicated a promising released profile. It can be concluded that the preparation technique could affect the release profile of drugs from nanofibers and the nanofibers with the core-shell structures mostly possess a better release profile compared to the mixed and sandwich structures. Based on the obtained results, the G6 can be considered as effective formulation on ophthalmic sustained drug delivery of GNT and MP, while G7 can be known as a suitable formulation for short-term delivery of GNT.

## Acknowledgments

The authors would like to acknowledge the Research Council of Kermanshah University of Medical Sciences (Grant number: 96650) for financial support of this work. Also, faithfully thank Rahesh Daru Novin (RDN) knowledge-based company for cooperation in providing materials and equipment.”

## Ethical Issues

The whole procedure was approved by the Ethics Committee (approval number: IR.KUMS.REC.1396.548), Kermanshah University of Medical Sciences, Kermanshah, Iran.

## Conflict of Interest

There is no conflict of interest.
